# The use of immunotherapy in the treatment of melanoma

**DOI:** 10.1186/s13045-017-0458-3

**Published:** 2017-04-24

**Authors:** Tala Achkar, Ahmad A. Tarhini

**Affiliations:** 1 University of Pittsburgh, Pittsburgh, PA USA; 2 University of Pittsburgh Cancer Institute, 5150 Centre Avenue, Room 555, Pittsburgh, PA 15232 USA

**Keywords:** Melanoma, Immunotherapy, Anti-CTLA4, Anti-PD1

## Abstract

Patients with advanced melanoma have a compromised anti-tumor immune response leading to tumor immune tolerance and a tumor microenvironment conducive to disease progression. Immunotherapy that successfully overcomes this tumor-mediated immune suppression has made the greatest impact in the management of this disease over the past few years. This progress through immunotherapy builds upon earlier successes that interferon-α had in the treatment of melanoma in the adjuvant setting, as well as that of high-dose interleukin-2 in advanced melanoma. The development of immune checkpoint inhibitors has led to dramatic clinical activity in advanced melanoma. In particular, anti-CTLA4 and anti-PD1 monoclonal antibodies have taken us forward into the realm of longer survival and durable responses with the possibility of cure in a continuously increasing proportion of patients. Combination immunotherapeutic strategies and novel immunotherapeutic agents are being tested at an accelerated pace where the outlook for long-term survival benefits for the majority of patients appears brighter than ever.

## Background

The incidence of melanoma has been increasing such that it is now the fifth and seventh most common cancer among men and women, respectively, in the USA [1]. Specifically in the USA, the Surveillance, Epidemiology and End Results (SEER) data shows that among Caucasians, there has been a 60% increase in incidence over the last 30 years [2]. For many years, there has continued to be a high rate of death from metastatic melanoma with an estimated 10,130 deaths from melanoma in 2016 [3]. There has been a recent change in our ability to control and treat metastatic melanoma as a result of our better understanding of immunology and development of immunotherapy [4, 5]. In this review, we aim to discuss the development and application of immunotherapy in the clinical practice of advanced melanoma treatment.

## Adjuvant therapy for high-risk resected melanoma

Interferon-alfa (IFNα) exerts its effects via different mechanisms including immunoregulatory, anti-angiogenic, differentiation-inducing, anti-proliferative, and pro-apoptotic [6]. It also acts to promote tumor immunogenicity by enhancing dendritic cell (DC) response to the tumor, as well as DC maturation and antigen presentation that contribute to anti-tumor immunity [6–8]. This shift in host immunity occurs by shifting from a Th2 predominant response to a Th1 response, thereby leading to amplification of cell-mediated cytotoxicity and increased Th1 lymphocytes in the tumor environment [9–16].

### High-dose IFNα

High-dose IFNα (HDI) is the standard of care in the adjuvant setting for the treatment of resected stage IIB/III melanoma. In randomized controlled trials evaluating various doses of IFNα in the adjuvant treatment of high-risk melanoma (stages IIB, III, or IV), a durable impact on both relapse-free survival (RFS) and overall survival (OS) was only seen with the regimen utilizing HDI as tested in Eastern Cooperative Oncology Group (ECOG) and US Intergroup trials E1684 (*n* = 287; significant RFS and OS benefit vs. observation), E1690 (*n* = 642; only RFS benefit seen vs. observation), and E1694 (*n* = 880; significant RFS and OS benefit vs. vaccine) [17–19]. These studies used a HDI regimen that was administered first as a 4-week induction phase, with IFNα given at a dose of 20 million IU/m^2^/day intravenously for five consecutive days every week. This induction phase was followed by a maintenance phase of subcutaneous IFNα at a dose of 10 million IU/m^2^/day every other day three times each week for an additional 48 weeks.

All three phase III trials (E1684, E1690, and E1694) showed significant improvement in RFS; however, there was a significant improvement in OS only in E1684 and E1694. E1684 reported a median OS of 3.82 vs. 2.78 years (*P* = 0.0237) in the HDI group compared with observation, at a median follow-up of 6.9 years. There were also significant improvements in RFS with a median RFS of 1.72 years vs. 0.98 years in the HDI group compared with observation (*P* = 0.0023) [17]. This trial led to the FDA approval of HDI in 1995. In E1694, HDI was compared with a ganglioside vaccine (GMK; ganglioside conjugate vaccine coupled to keyhole limpet hemocyanin with QS-21 as adjuvant) and demonstrated significant RFS benefit (HR 1.47; *P* = 0.0015) as well as OS benefit (HR 1.52; *P* = 0.009) in the HDI arm compared with the GMK vaccine at a median follow-up of 16 months [19].

In E1690, the HDI regimen described above was used, in addition to a low-dose regimen of IFNα (LDI; dose of 3 million units SC 3×/week for 2 years). These were compared to observation. In the HDI arm, the 5-year estimated RFS rate was 44% (*P* = 0.03), and this was the only arm to reach statistical significance for RFS [18]. Neither HDI nor LDI demonstrated an OS benefit compared to observation (52% HDI arm vs. 53% LDI arm vs. 55% observation arm). Of note, when the E1690 observation arm was compared to the E1684 observation arm, the E1690 arm had a higher OS (median 6 vs. 2.8 years), and the subjects in E1690 were not required to undergo a lymph node dissection unlike those in E1684. Additionally, a retrospective analysis of E1690 revealed that surgical intervention followed by IFN therapy in relapsing subjects in the observation group might have impacted the survival analysis in this study.

### Pegylated IFNα

Pegylated IFNα (Peg-IFN) is created by covalent bonding of the IFN molecule with polyethylene glycol resulting in a compound with sustained absorption and a longer half-life. Peg-IFN was tested in EORTC 18991 and was approved in the USA in 2011 for use as adjuvant therapy in patients with high-risk melanoma with lymph node metastases [20]. The EORTC 18991 trial investigated the efficacy and safety of Peg-IFN in patients with resected AJCC stage III melanoma as compared to observation. Peg-IFN was administered first as an induction dose of 6 mcg/kg once weekly for 8 weeks, followed by maintenance doses of 3 mcg/kg once weekly for up to 5 years. At a median follow-up of 7.6 years, there was an improved RFS in the Peg-IFN arm (HR 0.87; 95% CI 0.76–1.00; *P* = 0.05), but there was no difference in OS or in distant metastasis-free survival (DMFS) between the two arms. Patients with microscopic nodal metastases and ulcerated primary tumors had the greatest improvement in RFS, OS, and DMFS. Importantly, during the study, Peg-IFN was discontinued in 37% as a result of toxicity.

### Adjuvant ipilimumab

Cytotoxic T lymphocyte antigen-4 (CTLA-4) blockade with ipilimumab was tested in the adjuvant setting. The phase III EORTC 18071 trial (*n* = 951) randomized stage III melanoma patients following complete surgical resection in a 1:1 fashion to receive ipilimumab given at 10 mg/kg or to placebo. In the absence of disease relapse or limiting toxicities, ipilimumab was given intravenously every 3 weeks for up to 4 doses (induction) then every 3 months for up to 3 years of maintenance [20]. After a median follow-up of 2.7 years, there was a 46.5 vs. 34.8% DFS in patients in the ipilimumab vs. placebo arms (*P* = 0.0013). Of note, in patients receiving ipilimumab, immune-related grade 3/4 adverse events (AEs) included gastrointestinal (16%), endocrine (8.5%), and liver toxicities (11%). Discontinuation due to AEs occurred in 52% of patients in the ipilimumab group, including 39% during the induction phase. Death due to drug-related AEs occurred in five patients (1%). Overall survival data from this study were presented in October 2016 at the 2016 European Society for Medical Oncology (ESMO) meeting revealing significant improvement with ipilimumab, HR 0.72 (0.58, 0.88), *p* = 0.001 [21].

### Ongoing adjuvant clinical trials

The randomized controlled phase III trial E1609 is comparing standard HDI to ipilimumab in patients with surgically resected stage IIIB, IIIC, M1a, and M1b melanoma. Ipilimumab was given at two different doses: 3 or 10 mg/kg tested separately in comparison with HDI [NCT01274338]. The findings of E1609 will add important information on the clinical efficacy of adjuvant ipilimumab vs. HDI, as well as provide data regarding the lower and less toxic dose of ipilimumab (3 mg/kg) which is the standard for advanced inoperable metastatic melanoma.

Clinical trials testing adjuvant therapy with the anti-PD1 antibodies pembrolizumab and nivolumab are ongoing. US Intergroup S1404 is testing pembrolizumab at 200 mg IV every 3 weeks versus the choice of HDI or ipilimumab at 10 mg/kg in patients with resected stage III/IV melanoma [NCT02506153]. KEYNOTE-054 is testing pembrolizumab versus placebo in patients with resected stage III disease [NCT02362594]. CheckMate 238 is testing nivolumab versus ipilimumab at 10 mg/kg in patients resected stage IIIB/C or IV melanoma [NCT02388906].

## High-dose interleukin-2 in the treatment of metastatic melanoma

Interleukin-2 (IL-2) is produced by antigen-stimulated CD4+ T cells and, to a lesser extent, by CD8+ T cells, NK cells, and activated dendritic cells (DCs) [22, 23]. IL-2 not only augments an effector lymphocyte immune response but also is an immune regulator and expands immunosuppressive CD4+ FOXP3+ T regulatory cells (Treg) [24]. It also serves to promote activation-induced death (AICD) of over-activated T cells [25]. As such, the administration of IL-2 results in an abundant release of pro-inflammatory cytokines (including TNF-α, IL-1β, IL-6, IFN-γ) which is likely the underlying cause of the “flu-like” side effects of treatment. The capillary leak syndrome (CLS) and hypotension observed in patients receiving high-dose IL-2 is likely a result of the increase in angiopoietin 2 and nitric oxide levels [26, 27].

IL-2 is administered as a high-dose bolus (HDB) using doses of 600,000–720,000 units/kg every 8 h on days 1 to 5 (cycle 1) and on days 15 to 19 (cycle 2). A maximum of 14 doses are given per cycle, or 28 doses are given per course (2 cycles). In studies, IL-2 was either administered as a single agent or in combination with immunologically active cells [28]. This latter technique is known as adoptive immunotherapy and uses two types of immune cells: lymphokine-activated killer (LAK) cells and tumor-infiltrating lymphocytes (TIL). In seven phase II trials including 255 patients with renal cell carcinoma (RCC) receiving HDB IL-2, an overall response rate of 15% was seen [29]. The median duration of response for partial responders (PR) was 19 months while the duration of response for complete responders (CR) was not reached.

In metastatic melanoma, a retrospective analysis of eight trials using the HDB IL-2 regimen which included 270 patients demonstrated an objective response rate of 16% [30]. The median response duration was 8.9 months (4–106+ months). Of the patients who responded, 28% (including 59% of those patients who achieved a complete response) remained progression free at a median follow-up of 62 months. There were no relapses among patients who had an ongoing response at 30 months, and follow-up extended beyond 20 years in some cases suggesting that these patients are likely cured. The major toxicities associated with HDB IL-2, including CLS leading to hypotension, renal insufficiency, and hypoxia, have prevented the widespread application of this therapy. Its use has been limited to specialized programs with experienced staff and is generally only offered to patients with good performance status and organ function [31].

Randomized studies have not shown improved outcome for IL-2 administered with LAK cell compared with HDB IL-2 alone; however, other efforts in adoptive immunotherapy including the simplification and harvesting of TIL are leading to significant advances [32–34]. The infusion of ex vivo expanded TIL after chemotherapy-induced lymphodepletion or total body radiotherapy in conjunction with HDB IL-2 has resulted in response rates of 50–72% in selected patients with successful TIL harvesting and expansion [34, 35].

## Inhibitors of immune checkpoints

One of the most successful immunotherapeutic strategies to enhance the anti-tumor response has been the use of monoclonal antibodies that block immunoregulatory mechanisms that suppress host responses to tumor antigens. We will review these antibodies in the upcoming sections.

### Blockade of cytotoxic T lymphocyte antigen-4

CTLA-4 is a member of the CD28:B7 immunoglobulin superfamily. It is normally expressed at low levels at the surface of naïve effector T cells and regulatory T cells (Tregs). After naïve T cells are stimulated via the T cell receptor, CTLA-4 localizes to the plasma membrane where it competes with CD28 for B7, which ultimately turns off T cell receptor signaling [36]. As such, under physiologic conditions, CTLA-4 serves as a critical check point inhibitor as it downregulates T cell activation to prevent autoimmunity and allow tolerance to self-antigens [37].

Ipilimumab and tremelimumab are anti-CTLA-4 monoclonal antibodies that cause blockade of CLTA-4 signaling resulting in prolonged T cell activation, T cell proliferation, and an amplification of T cell-mediated immunity leading to an enhanced anti-tumor immune response [37, 38].

#### Ipilimumab

Ipilimumab has been approved by the FDA for the treatment of advanced melanoma. Two important phase III trials with ipilimumab in advanced inoperable AJCC stage III and stage IV melanoma have been completed in both the first-line and second-line settings. The first-line trial compared first-line treatment of combination therapy of ipilimumab at 10 mg/kg and dacarbazine (DTIC) versus dacarbazine and placebo. The results showed an OS that was significantly longer in previously untreated patients receiving ipilimumab and DTIC compared to those who received DTIC and placebo (11.2 versus 9.1 months; HR 0.72; *P* < 0.001). There were higher survival rates in the ipilimumab and DTIC group at 1 year (47.3 versus 36.3%), 2 years (28.5 versus 17.9%), and 3 years (20.8% versus 12.2%) [39].

The second trial compared ipilimumab (3 mg/kg) with or without the gp100 peptide vaccine versus the gp100 peptide vaccine alone in the second-line setting. Ipilimumab was given IV at 3 mg/kg every 3 weeks for four doses. A total of 676 pre-treated patients were randomized, and responding patients were eligible for re-induction with ipilimumab if they relapsed. The 1- and 2-year survival rates were 44% (ipilimumab + gp100), 46% (ipilimumab + placebo), and 25% (ipilimumab + placebo) and 22% (ipilimumab + gp100), 24% (ipilimumab + placebo), and 14% (gp100 + placebo), respectively. The best objective response rate was 5.7% (ipilimumab + gp100), 10.9% (ipilimumab + placebo), and 1.5% (gp100 + placebo). Median OS increased from 6.4 to 10.0 months with the addition of ipilimumab to gp100 vaccine (HR 0.68; *p* < 0.0001) [40].

Importantly, in a recent analysis of 1861 melanoma patients treated with ipilimumab in clinical trials, 21% were still alive at 3 years with survival plateauing with a maximum follow-up of about 10 years [41]. Ipilimumab demonstrated survival benefits in metastatic melanoma that had never been achieved with any prior treatments. Simultaneously, CTLA-4 blockade presented new challenges in the diagnosis and management of immune-mediated toxicities related to the mechanism of action of ipilimumab, leading to adverse events that can be life-threatening and may require treatment with systemic corticosteroids and/or other immunosuppressants [42].

#### Tremelimumab

Tremelimumab has similarly shown promising clinical activity in advanced melanoma at initial testing leading to a phase III clinical trial (A3671009) in patients with treatment-naïve metastatic melanoma. This open-label study randomized patients to therapy with single-agent tremelimumab at 15 mg/kg IV every 12 weeks (*n* = 328) or standard-of-care chemotherapy (*n* = 327) with either dacarbazine or temozolomide with a primary endpoint of overall survival [43]. This trial was closed for “futility” after a second interim analysis when the log-rank test statistic (*p* = 0.729) crossed the pre-specified O’Brien-Fleming futility boundary. It remains notable that the 1-year survival rate for tremelimumab was >50% and the median survival was 12.02 months (compared to 10.45 months for chemotherapy). Furthermore, the majority of the responses to tremelimumab were durable. Factors that may have affected the survival analysis of this trial are eligibility criteria (limited by LDH), availability of alternate anti-CTLA4 strategies to which patients had access, and the salvage patterns of patients on the chemotherapy arm.

### PD-1 and PD-L1 as immunotherapeutic targets for melanoma

Programmed cell death-1 (PD-1) is an immune-inhibitory receptor that belongs to the CD28/CTLA4 receptor family [44–47]. PD-1 binds to two known ligands PD-L1 (B7-H1) [44–48] and PD-L2 (B7-DC) which are widely expressed in a variety of tissues [49, 50]. Once PD-1 binds to PD-L1, it negatively regulates T cell functions [45–48].

PD-L1 is expressed in many tumors, including melanoma [51, 52]. PD-1/PD-L1 interactions have been studied in animal models, as well as in vitro, and they have been shown to inhibit the effector functions of tumor-specific CD8+ T cells, thereby contributing to tumor-induced immunosuppression leading to tumor resistance to cytotoxic T cell responses [51–53].

High expression of PD-L1 on tumor cells has been found to correlate with poor prognosis and survival in various cancer types, including renal cell carcinoma (RCC), ovarian carcinoma, and melanoma [54–56]. However, more recently, studies have shown that expression of PD-L1 metastatic melanoma correlates with the presence of tumor-infiltrating lymphocytes (TILs) in human melanocytic lesions, such that 98% of PD-L1(+) tumors were associated with TILs compared with only 28% of PD-L1(−) tumors. PD-L1(+) melanocytes were almost always localized immediately adjacent to TILs [57]. Interestingly, IFN-γ, a primary inducer of PD-L1 expression, was detected at the interface of PD-L1(+) tumors and TILs suggesting that TILs trigger their own inhibition by secreting cytokines that drive tumor PD-L1 expression. Consistent with this hypothesis, overall survival of patients with PD-L1(+) metastatic melanoma was significantly longer than patients with PD-L1(−) metastatic melanoma [57]. Multiple anti-PD-1 monoclonal antibodies are currently in use and have shown promising activity in the management of advanced melanoma.

#### Pembrolizumab

Pembrolizumab is a humanized monoclonal antibody (IgG4/kappa isotype) that blocks the interaction between PD-1 and its ligands, PD-L1 and PD-L2. It was evaluated in an open-label phase I trial (KEYNOTE-001), which initially evaluated three different doses: 1, 3, and 10 mg/kg administered every 2 weeks. All three doses were tolerated, and given that pembrolizumab has a half-life of 21 days, the protocol changed the dosing frequency to every 3 weeks. Patients with advanced melanoma who were ipilimumab naïve (*n* = 179) and ipilimumab treated (*n* = 115) were enrolled and given pembrolizumab at 10 mg/kg (*n* = 183) or 2 mg/kg (*n* = 111) [58]. The overall response rate was 34%: 44% in the treatment-naïve, 40% in the ipilimumab-naïve, and 28% in the ipilimumab-treated patients. These responses were durable, and the median duration of response was not reached (6–76+ weeks). Median progression-free survival (PFS) was 5.5 months, and OS was 69% at 1 year [59]. Of note, response rates and PFS were significantly higher in patients who had high PD-L1 tumor expression [60]. A 3-year OS update was presented at the 2016 ASCO Annual Meeting and included 655 patients who were enrolled and treated on this trial. There was a 40% 3-year OS rate in all patients including 45% OS rate in treatment-naïve patients [61]. FDA approval of pembrolizumab in September 2014 at the dose of 2 mg/kg every 3 weeks was granted based on initial data analysis from a cohort of the phase I trial in which 173 patients received pembrolizumab 2 mg/kg (*n* = 89) or 10 mg/kg (*n* = 84) every 3 weeks and covered pretreated patients. The label was later expanded to include treatment-naïve patients as later data became available [62].

The phase II clinical trial, KEYNOTE-002, evaluated two doses of pembrolizumab (2 or 10 mg/kg) compared with the investigator’s choice of chemotherapy in patients with advanced melanoma [63]. Both the 2 and 10 mg/kg doses of pembrolizumab had improved PFS over chemotherapy (HR 0.57; 95% CI 0.45–0.73; *p* < 0.0001 and HR 0.50; 95% CI 0.39–0.64; *p* < 0.0001, respectively) across all subgroups, as well as a higher overall response rate compared to chemotherapy (21 and 25 vs. 4%).

Pembrolizumab was also evaluated in a multicenter, randomized phase III trial (KEYNOTE-006), which compared two different dosing schedules (10 mg/kg every 2 weeks or every 3 weeks) with ipilimumab [64]. The overall response rate (ORR) was 33% (pembrolizumab) vs. 12% (ipilimumab). The PFS after 6 months of treatment was 45% for the pembrolizumab arms and 26% for the ipilimumab arm, with an OS of 87 versus 75%. At 12 months, OS rates were 74% (every 2 weeks) and 68% (every 3 weeks) for the two pembrolizumab arms and 58% for the ipilimumab arm. Finally, pembrolizumab was superior to ipilimumab in this study in all subset analyses of pre-specified groups, including PD-L1(+) and PD-L1(−) groups. Final OS analysis was presented at the 2016 ASCO Annual Meeting. ORR was 36–37% in the pembrolizumab groups (12–13% CR) versus 13% (5% CR) in the ipilimumab groups. At a median follow-up of 23 months, median OS was not reached for pembrolizumab. At 24 months, 55% of pembrolizumab-treated patients overall were alive, including approximately 30% who were alive and progression free [65].

#### Nivolumab

Nivolumab is a fully human anti-PD-1 monoclonal antibody (IgG4). In studies evaluating efficacy and safety, nivolumab was given at various doses ranging from 0.1 to 10 mg/kg. It was tolerated at up to 10 mg/kg, which is the highest dose tested, and no maximum tolerated dose was identified [66]. The 10 mg/kg dose of nivolumab had more high grade 3/4 drug-related adverse events (AEs) than the other doses, although the spectrum, frequency, and severity of AEs was generally similar across all doses. The incidence of immune-related AEs (irAEs) was approximately 20% and included pruritus, rash, and diarrhea. Other irAEs include increase of TSH, increase of ALT/AST, pneumonitis, infusion reaction, and vitiligo.

In a phase I trial of nivolumab in ipilimumab-naïve patients with advanced melanoma, the median OS was 17.3 months (all doses) and 20.3 months at the 3 mg/kg dose. Survival rates were 63% at 1 year, 48% at 2 years, and 41% at 3 years. Median PFS was 3.7 months across doses and 9.7 months at 3 mg/kg [67]. Based on safety data and further studies (including CheckMate 037), nivolumab is given at a dose of 3 mg/kg every 2 weeks in subsequent trials and became the second monoclonal antibody against PD-1 receptor to be approved by the FDA for the treatment of patients with unresectable or metastatic melanoma and disease progression following ipilimumab and a BRAF inhibitor (if *BRAF* V600 mutation positive).

CheckMate 037 was a phase III trial on patients with metastatic melanoma who progressed on or after anti-CTLA-4 therapy and a BRAF inhibitor (if *BRAF* V600 mutation positive) which demonstrated the efficacy of nivolumab compared to the investigator’s choice of chemotherapy, with an overall response rate (ORR) of 32 vs. 11% [68]. Nivolumab also demonstrated significant efficacy in ipilimumab-naïve patients with advanced melanoma [69]. Long-term follow-up in the phase I study of nivolumab determined 2-year and 3-year overall survival rates of 48 and 41%, respectively, with nivolumab when given to treatment-naïve patients [70].

The combination of ipilimumab and nivolumab given concurrently or sequentially was evaluated in a phase I study, and depending on the dose, the combination resulted in response rates of approximately 50% with many durable responses [71]. Updated data from this trial demonstrated that concurrent treatment with nivolumab and ipilimumab resulted in a 2-year survival rate of 79% [72]. However, there was a 62% rate of grade 3/4 irAEs at the optimal doses.

CheckMate 069 was a randomized phase II double-blind trial with 142 patients with metastatic melanoma who are treatment-naïve patients [73]. Patients were assigned in a 2:1 fashion to ipilimumab (3 mg/kg) combined with either nivolumab (1 mg/kg) or placebo every 3 weeks for four doses, followed by nivolumab (3 mg/kg) or placebo every 2 weeks until disease progression or toxic side effects. Patients with BRAF wild-type tumors had an objective response rate of 61% in the combination group versus 11% in the ipilimumab monotherapy group (*p* < 0.001). Furthermore, there were complete responses in 22% of the patients in the combination group and none in the ipilimumab monotherapy group. Median PFS was not reached in the combination therapy group and was 4.4 months in the ipilimumab group (HR 0.40; 95% CI 0.23 to 0.68; *p* < 0.001). Similar results were also seen in patients with BRAF mutation-positive tumors. In a later update with a median follow-up of 24.5 months, the 2-year overall survival rate in the combination arm was 63.8% (95% CI 53.3–72.6) and 53.6% (95% CI 38.1–66.8) for those with ipilimumab alone [74].

CheckMate 067 was a phase III double-blind study comparing nivolumab plus ipilimumab to nivolumab alone and to ipilimumab alone in treatment-naïve patients (*n* = 945) with advanced melanoma. The ORR with nivolumab alone was 43.7%, in combination with ipilimumab was 57.6%, and ipilimumab monotherapy was 19% [75]. Treatment-related AEs were more frequently seen in the combination group (grade 3/4, 55%) than with nivolumab (grade 3/4, 16%) or with ipilimumab alone (grade 3/4, 27%).

## Other immune checkpoints as immunotherapeutic targets

### CD40

CD40 is a co-stimulatory molecule that is a member of the tumor necrosis factor (TNF) superfamily, which is involved in the regulation of immune function. It is widely expressed by immune cells as well as cancer cells and has been implicated in the regulation of humoral and cellular immunity as well as pro-apoptotic and anti-proliferative activity [76–79]. CD40 is expressed on dendritic cells and is activated by the CD40 ligand which is found on activated T cells. This interaction leads to T cell activation, and in CD40, deficient tumors result in the induction of systemic cytotoxic T lymphocyte immunity [80, 81].

CP-870,893 (Pfizer) is a fully human IgG2 agonist monoclonal antibody that targets CD40. In a phase I study of intravenous infusions in 29 patients, the maximum tolerated dose (MTD) was estimated to be 0.2 mg/kg, with a dose-limiting cytokine-release syndrome characterized by fevers, chills, and rigors. Notably, melanoma antigen-specific T cells were induced, and objective partial responses were noted in four patients with metastatic melanoma [82].

Following this, a phase I trial of weekly dosing of CP-870,893 for up to eight doses was conducted in 27 patients. The MTD was again estimated to be 0.2 mg/kg limited by a cytokine-release syndrome [83].

Dacetuzumab (SGN-40) is a humanized IgG1 agonist monoclonal antibody that also targets CD40 [84]. A phase I single-dose study in patients with lymphoid malignancies, acute myeloid leukemia, and multiple myeloma demonstrated safety up to 6 mg/kg with no MTD declared [85].

Dacetuzumab has been evaluated in patients with relapsed/refractory DLBCL with an ORR of 9%. Common non-hematologic adverse events (AEs) included fatigue, headache, chills, fever, and nausea. The most frequently observed grade 3/4 non-hematologic AE was deep venous thrombosis (three patients). The promising results from early clinical trials have encouraged further drug development in order to investigate the effect of CD40 monoclonal antibodies in combination with other cancer immunotherapies.

### OX40

OX40 and its ligand, OX40L, are members of the TNF family. They lead to T cell expansion, cytokine production, and cell survival. OX40 is expressed transiently on CD4+ and CD8+ activated T cells as well as CD4+ CD25+ Tregs and controls Treg differentiation and suppressive function. Activation of OX40 on Tregs appears to stop their suppressive function [86–88].

In mouse models, OX40 interaction with OX40L during tumor priming demonstrated anti-tumor activity [89]. A murine agonist monoclonal antibody against OX40 was tested in a phase I trial and had acceptable toxicity resulting in five of 20 patients with stable disease [90].

### CD137

The CD137 receptor and its ligand are members of the TNF family. The interaction between the CD137 receptor, expressed on activated T cells, and ligand stimulates T cell activities and enhances T cell proliferation as well as the memory and cytotoxic activity of T cells [91–93].

BMS-663513 is a fully human anti-CD137 agonist monoclonal antibody that has been tested in a phase I dose escalation study with 83 patients (54 melanoma, 15 RCC, 13 ovarian, and 1 prostate) [94]. Three patients responded to treatment, and four had stable disease.

## Intralesional immunotherapy

The goal of intralesional therapy is local tumor regression in the injected metastases as well the induction of systemic immune responses. Talimogene laherparepvec (T-VEC) is an oncolytic immunotherapy which consists of a herpes simplex virus type 1 (HSV1) backbone that contains the gene for GM-CSF. A phase III trial tested T-VEC compared to GM-CSF in patients with stage IIIB–IV melanoma [95]. There was a 16.3% durable response rate with T-VEC, as well as an ORR of 26.4%. The median survival in the T-VEC group was 23.3 months compared to 18.9 months in the GM-CSF group (*p* = 0.051) [95].

Overall, intralesional approaches have been shown to be relatively safe and well tolerated with evidence of local and bystander/distant anti-tumor activity. This therapy is promising and can be combined with other immune-activating agents such as cytokines and checkpoint inhibitors. Combination studies of T-VEC with anti-CTLA4 and anti-PD1 antibodies are underway in metastatic disease.

## Adoptive cell therapy

Adoptive cell therapy (ACT) utilizes TIL that are harvested from a patient’s own tumor, which then undergo ex vivo expansion, lymphodepletion, and then are infused back into the patient. This regimen is typically followed by HDB IL-2 [96]. Multiple single-institution studies in patients with metastatic melanoma have demonstrated response rates that approach 50% with ACT, as well as complete response rates (CRs) in about 20% of patients, most of which have been durable CRs [34, 97, 98]. The need for expertise in TIL processing and cultures and the need for local specialized facilities have precluded the widespread use of ACT, but there are extensive efforts directed at making this form of immunotherapy more widely available such as the adoption of central processing facilities.

## The role of biomarkers predictive of therapeutic benefit

Predicting which patients will benefit from a particular treatment and which ones will not, thereby sparing patients adverse events and high cost of treatment, has led to great interest in the development of predictive biomarkers. Biomarkers that have been studied include gene expression signatures [99, 100], exome sequencing studies [101], and T cell expression patterns within the tumor microenvironment [63]. There are extensive efforts in progress focused on determining the utility of these biomarkers.

## Conclusions

Advances in the treatment of melanoma have focused on overcoming tumor-induced immune suppression and were initially established in the adjuvant setting with the use of IFN-α and HDB IL-2 in the treatment of metastatic disease. Further development of checkpoint inhibitors directed against CTLA-4 and PD-1 have demonstrated impressive clinical outcomes in the treatment of metastatic melanoma. Studies are continuing to evaluate combination immunotherapy regimens including nivolumab and ipilimumab, IFN-α and ipilimumab, and multiple anti-PD1/PDL1-based combination studies [71, 102]. Studies of other immune checkpoint modulators including CD40, OX40, and CD137 among others are also ongoing [5].

## Abbreviations

AE: Adverse event
AICD: Activation-induced death
CLS: Capillary leak syndrome
CR: Complete responders
CTLA-4: Cytotoxic T lymphocyte antigen-4
DC: Dendritic cells
DMFS: Distant metastasis-free survival
ECOG: Eastern Cooperative Oncology Group
ESMO: European Society for Medical Oncology
HDB: High-dose bolus
HDI: High-dose interferon-alfa
IFNα: Interferon-alfa
IL-2: Interleukin-2
irAEs: Immune-related adverse events
LAK: Lymphokine-activated killer
ORR: Overall response rate
OS: Overall survival
PD-1: Programmed cell death-1
Peg-IFN: Pegylated IFNα
PFS: Progression-free survival
RCC: Renal cell carcinoma
RFS: Relapse-free survival
SEER: Surveillance, Epidemiology, and End Results
TIL: Tumor infiltrating lymphocytes
TNF: Tumor necrosis factor
Treg: T regulatory cells
